# Supplementary material for: Efficacy of Licensed Monoclonal Antibodies and Antiviral Agents against the SARS-CoV-2 Omicron Sublineages BA.1 and BA.2
Source: Viruses. 2022 Jun 23;14(7):1374. doi: 10.3390/v14071374 (PMC9321742; doi:10.3390/v14071374)
Supplement: Supplementary file 1 [file viruses-14-01374-s001.zip › viruses-1766348-supplementary.pdf]

Supplementary Table S1. Lineage classification of strains included in the study, accession number and spike identified mutations.

Code	Pango Lineage	WHO	Spike aa Substitutions
**EPI_ISL_2472896**	B.1		D614G
**EPI_ISL_2840619**	B.1.617.2	Delta	T19R, E156G, F157del, R158del, S255F, L452R, T478K, D614G, P681R, D950N
**EPI_ISL_8020894**	BA.1.17	Omicron	A67V, D614G, D796Y, E484A, F220del, G142D, G219del, G339D, G446S, G496S, H69del, H655Y, ins152KSW, ins214EPE, K417N, L212I, L981F, N211del, N440K, N501Y, N679K, N764K, N856K, N969K, P681H, Q493R, Q498R, Q954H, S221del, S371L, S373P, S375F, S477N, T95I, T478K, T547K, V70del, V143del, Y144del, Y145del, Y505H
**EPI_ISL_11814364**	BA.2.13	Omicron	A27S, D405N, D614G, D796Y, E484A, G142D, G339D, Spike H655Y, K417N, L24del, N501Y, N679K, N764K, N969K, P25del, P26del, P681H, Q493R, Q498R, Q954H, R408S, S371F, S373P, S375F, S477N, T19I, T376A, T478K, V213G, Y505H

Supplementary Table S2. Clinical features of enrolled individuals.

ID	Sex	Age (Years)	mAbs	Symptoms	Comorbidities	Vaccination
**1**	Male	22	BAM/ETE	Fever, cough, headache	Myotonic Dystrophy Type 1, hypothyroidism	no
**2**	Male	70	BAM/ETE	Cough, gastrointestinal, dysgeusia/anosmia	Hypertension, dyslipidaemia, Type 2 Diabetes	no
**3**	Male	62	BAM/ETE	Headache, arthomyalgia	Hemosiderosis, previous HBV infection, other respiratory diseases	no
**4**	Male	66	BAM/ETE	Fever, cough, headache	Hypertension, dyslipidaemia, other respiratory diseases	no
**5**	Male	80	BAM/ETE	Fever	Hypertension, cardiopathy, haemato-oncological disease	yes
**6**	Female	80	BAM/ETE	Fever	Hypertension, BPCO, haemato-oncological disease, other respiratory diseases	yes
**7**	Female	37	BAM/ETE	Fever, cough, gastrointestinal, dysgeusia/anosmia, headache, arthomyalgia	BMI > 35	no
**8**	Female	67	BAM/ETE	Fever, arthomyalgia	Haemato-oncological disease, dyslipidaemia, latent tuberculosis, dysthyroidism	yes
**9**	Male	67	BAM/ETE	Fever, cough	Hypertension, cardiopathy, hypothyroidism, dyslipidaemia	no
**10**	Female	53	BAM/ETE	Fever, cough	Hypertension, Type 2 Diabetes	no
**11**	Male	40	BAM/ETE	Dysgeusia/anosmia, headache	Hypertension, dyslipidaemia, hyperuricemia	no
**12**	Female	52	BAM/ETE	Fever, cough, gastrointestinal, headache, arthomyalgia	Hypertension, BMI > 35	no
**13**	Female	77	CAS/IMD	Headache	Hypertension	no
**14**	Female	73	CAS/IMD	Cough	Hypertension, Type 2 Diabetes, COPD, hypothyroidism	no
**15**	Male	74	CAS/IMD	Dyspnoea	Hypertension, dyslipidaemia	no

**16**	Female	65	CAS/IMD	Fever, cough, dyspnoea, headache, arthomyalgia	Cardiopathy, other respiratory diseases	no
**17**	Female	77	CAS/IMD	Cough	Hypertension, cardiopathy, BMI > 35	no
**18**	Female	46	CAS/IMD	None	Hypertension, dyslipidaemia, BMI > 35	no
**19**	Female	88	CAS/IMD	Fever, cough	Hypertension, COPD	no
**20**	Male	62	CAS/IMD	Fever, cough, headache	Cardiopathy, Type 2 Diabetes, dyslipidaemia	no
**21**	Female	57	CAS/IMD	Cough, headache	Hypertension, autoimmune disease, hyperuricemia, BMI = 32	no
**22**	Male	18	CAS/IMD	Dysgeusia/anosmia	Cardiopathy	no
**23**	Male	77	CAS/IMD	Fever, cough	Hypertension, Chronic Ischemic Cardiomyopathy, cardiomyopathy, Type 2 Diabetes, haemato-oncological disease	yes
**24**	Male	75	CAS/IMD	Fever, cough	Hypertension, haemato-oncological disease	yes
**25**	Male	69	CAS/IMD	Fever, dysgeusia/anosmia, arthomyalgia	Hypertension, Chronic Ischemic Cardiomyopathy, dyslipidaemia	yes
**26**	Female	51	CAS/IMD	Dysgeusia/anosmia, gastrointestinal, cough, fever	BMI > 35	no
**27**	Female	54	SOT	Fever, cough, dysgeusia/anosmia, headache, arthomyalgia	No comorbidity	no
**28**	Female	52	SOT	Fever, cough	BMI > 35	no
**29**	Female	59	SOT	Fever, headache	Ischemic Chronic Cardiopathy, BMI > 35	no
**30**	Male	19	SOT	Fever, cough, headache, arthomyalgia	Other respiratory diseases	no
**31**	Female	34	SOT	Fever, arthomyalgia	Hemoglobinopathy	no
**32**	Male	56	SOT	Cough, gastrointestinal	Immunodeficiency	no
**33**	Male	78	SOT	Fever, cough, arthomyalgia	Cardiopathy	no
**34**	Female	83	SOT	Fever, arthomyalgia	Cardiopathy	no
**35**	Female	69	SOT	Cough, headache	Cardiopathy	no
**36**	Male	78	SOT	Fever, cough, headache	Chronic Ischemic Cardiomyopathy	no
**37**	Female	57	SOT	Fever, cough	Chronic Ischemic Cardiomyopathy, immunodeficiency	no
**38**	Female	55	SOT	Cough	Immunodeficiency	no
**39**	Male	52	SOT	Fever, arthomyalgia	Immunodeficiency	no
**40**	Male	41	SOT	Fever, cough	BMI > 35	no

BAM/ETE = Bamlanivimab plus etesevimab; CAS/IMD = casirivimab plus imdevimab; SOT = sotrovimab; BMI = body mass index; COPD = chronic obstructive pulmonary disease.

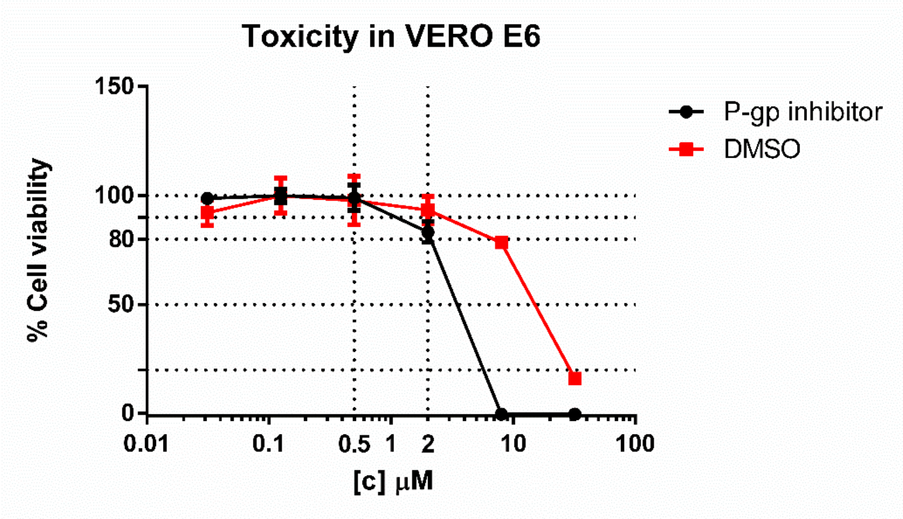

Supplementary Figure S1. Cytotoxicity of the P-gp inhibitor CP-100356 in VERO E6 cells. Cell viability was measured at 72h by CellTiter-Glo 2.0 Luminescent Cell Viability Assay (Promega). The luminescence values obtained from cells treated with P-gp inhibitor or DMSO were measured with the GloMax® Discover Multimode Microplate Reader (Promega) and elaborated with the GraphPad PRISM software version 6.01 (La Jolla, California, USA).
